# Effects of Concurrent Training on Oxidative Stress and Insulin Resistance in Obese Individuals

**DOI:** 10.1155/2015/697181

**Published:** 2015-02-04

**Authors:** Niara da Silva Medeiros, Fabiana Guichard de Abreu, Alana Schraiber Colato, Leandro Silva de Lemos, Thiago Rozales Ramis, Gilson Pires Dorneles, Cláudia Funchal, Caroline Dani

**Affiliations:** Centro Universitário Metodista IPA, 90420-060 Porto Alegre, RS, Brazil

## Abstract

Obesity is associated with insulin resistance (IR) and increased oxidative stress. Thus, the present study aimed to evaluate anthropometric parameters, IR, and oxidative stress in obese individuals subjected to two types of concurrent training at the same intensity but differing in frequency. Accordingly, 25 individuals were divided into two groups: concurrent training 1 (CT1) (5 d/wk) and concurrent training 2 (CT2) (3 d/wk), both with moderate intensity. Anthropometric parameters, IR, and oxidative stress were analyzed before and after 26 sessions of training. Both groups had reduced body weight and body mass index (*P* < 0.05), but only CT1 showed lower body fat percentage and increased basal metabolic rate (*P* < 0.05). Moreover, CT1 had increased HOMA-IR and decreased protein damage (carbonyl level), and CT2 had decreased HOMA-IR and increased lipid peroxidation (TBARS level) (*P* < 0.05). On the other hand, both training protocols reduced the GPx activity. It can be concluded that both types of concurrent training could be an alternative for lowering body weight and BMI. Also, it was observed that concurrent training, depending on the frequency, can contribute to reducing body fat, oxidative damage (protein oxidation), and IR but can induce oxidative damage to lipids. More studies are needed to elucidate the mechanisms involved.

## 1. Introduction

Sedentary lifestyle contributes to an increase in the incidence of obesity in most countries [1]. Obesity is defined as a chronic and multifactorial disease, which is associated with high mortality, especially in industrialized areas [1]. This disease is associated with many comorbidities, such as cardiovascular complications, hypertension, atherosclerosis, chronic inflammation, dyslipidemia, insulin resistance (IR), diabetes mellitus (DM), and other metabolic disorders [1, 2].

Furthermore, studies show that obesity can cause increased reactive species production and a depletion of antioxidant defenses, leading to oxidative stress. This condition induces oxidative damage to proteins, lipids, and DNA [3, 4]. Oxidative stress can be reduced by physical exercise with adequate frequency and intensity, which can provide adaptive changes to the regulation of antioxidant defenses, resulting in less oxidative cell damage [5–7].

The benefits of regular physical exercise are well documented [5]. However, there are few studies that approach the benefits of concurrent training (aerobic plus strength) in the obese population. Data demonstrate that this training can help to lose weight and body fat and to gain lean body mass [7, 8]. However, it is still unclear how concurrent training modulates parameters of IR and oxidative stress in obese individuals. Thus, this study aimed to evaluate anthropometric parameters, IR, and oxidative stress in obese individuals subjected to concurrent training of moderate intensity but differing in frequency.

## 2. Materials and Methods

### 2.1. Experimental Approach to the Problem

Concurrent training consisted in aerobic exercise combined with strength exercises. The training was divided into two groups: concurrent training 1 (CT1) and concurrent training 2 (CT2). In both training protocols, the participants were subjected to 26 sessions of 70 minutes each, with the same progressive intensity of 50–75% VO_2peak_ monitored by the Borg scale (Table 1). Each training session was divided into five minutes of initial warm-up, 30 minutes of walking, 30 minutes of strength exercises, and five minutes of stretching. CT1 was performed five days per week and CT2 three days per week. These different training frequencies were used to check for changes of oxidative profile and insulin resistance in obese sedentary individuals.

### 2.2. Subjects

The study was conducted in the Centro Universitário Metodista IPA, in southern Brazil. The study included individuals >18 years of age, sedentary (no more than 150 minutes of exercise per week) [9], with body mass index (BMI) of 30–40 kg/m^2^.

Thus, this study consisted of 25 individuals, 18 women and 7 men. The CT1 group was composed of 8 women and 4 men with age of 45.33 ± 10.46 years and height of 161.58 ± 6.19 cm. The CT2 group was composed of 10 women and 3 men, with age of 49.15 ± 9.47 years and height of 163.46 ± 10.43 cm. The two groups did not differ statistically in baseline values of age, height, and BMI (*P* > 0.05), indicating homogeneous groups.

The exclusion criteria were subjects who reported a history of autoimmune diseases, cancer, smoking, and diseases that make it impossible to engage in physical exercise and/or had contraindications. All participants read and signed an informed consent form and also had a doctor's permission to perform physical exercise. This study was approved by the Research Ethics Committee of the Centro Universitário Metodista IPA under protocol 37/12.

### 2.3. Procedures

Before the beginning of the training period, all individuals had a cardiorespiratory test, which consisted of a progressive test until exhaustion on a treadmill (ATL Inbramed Millennium, Porto Alegre, Brazil) with an ergospirometer (V02000, Medgraphics, St. Paul, Minnesota, USA). The test was conducted according to the modified Bruce protocol [9]. The Borg scale was used to monitor the intensity of the test, so that the intensity could be reproduced during physical training. To determine the intensity of exercise and follow a progressive linear intensity, the highest mean oxygen uptake (VO_2_) during 30 seconds was expressed as the peak oxygen uptake (VO_2peak_) because a VO_2_ plateau was invariably not observed during the test, although other criteria for VO_2max⁡_ given in the literature (i.e., RER > 1.1 and maximum HR within 10 beats of the age-appropriate reference value) were fulfilled [10].

To monitor the intensity of strength exercise, maximal dynamic strength was assessed by the one repetition maximum test (1RM) (following a progressive intensity of 50–75%) [11]. The 1RM test was performed for lower body by squat exercise and for upper body by supine exercise with free weights before starting the training period.

Strength training was based on the method of alternating segments with the following exercises: bench press with dumbbells plan, squats, shoulder abduction, plantar flexion, horizontal pulley, and abdominal crunch. During training, we applied progressive intensity (50–75% 1RM) as well as decreased volume of repetitions in all exercises, except for abdominal and plantar flexion. The abdominal and plantar flexion exercises were performed with no load and fixed in 15 repetitions.

Anthropometric measurements were assessed before and after the training period, including body weight (kg) (Welmy semianalytical balance), height (cm) (Welmy stadiometer), BMI (defined as the weight (kg) divided by height (m), squared kg/m^2^), abdomen, waist, and hip circumferences (cm), free fat mass (kg), body fat percentage (%), and basal metabolic rate (kcal) by the bioelectrical impedance method (Byodinamics BIA 310e).

Moreover, before and after the training period, samples of venous blood were collected (10 mL) without anticoagulant, after an overnight fast of 8 hours. Serum was separated by centrifugation at 1000 g for 10 minutes, aliquoted, and frozen at −20°C for further analysis.

We analyzed fasting glucose (automated method, COBAS C111, mg/dL), fasting insulin (DRG Insulin ELISA Kit, *μ*IU/mL), and HOMA-IR (homeostasis model of IR) calculated by the formula (fasting insulin (*μ*IU/mL) × fasting glucose (mmol/L)/22.5).

To evaluate the oxidative profile of the participants, lipid peroxidation was determined by the thiobarbituric acid reactive substances (TBARS) method described by Wills [12]. Protein carbonyl levels were measured to determine protein oxidation as described by Reznick and Packer [13]. Total sulfhydryl groups were assayed by the technique described by Aksenov and Markesbery [14]. We also determined the activities of the antioxidant enzymes catalase (CAT) [15], superoxide dismutase (SOD) [16], and glutathione peroxidase (GPx) [17].

### 2.4. Statistical Analysis

All variables were tested for normality of distribution by the Shapiro-Wilk test. For those that showed normality, we used the paired *t*-test for comparison of before and after training (mean ± standard error). All analyses were performed by the Statistical Package for Social Sciences (SPSS) program version 18.0. All statistical tests were two-tailed and performed using a significance level of *α* = 0.05.

## 3. Results

It was observed that both training protocols were able to reduce body weight and BMI (Table 2). But only CT1 showed significantly decreased body fat percentage and increased free fat mass and BMR after the training period. Regarding the circumference measurements, hip circumference was decreased in CT1, and abdomen and hip circumferences were decreased in CT2 (Table 2).

With regard to IR parameters, the CT1 group showed a significant increase in fasting insulin level and HOMA-IR and no change in fasting glucose. However, the CT2 group showed a significant decrease in fasting glucose level and HOMA-IR, with no change in fasting insulin (Table 3).

Finally, for oxidative stress markers, only carbonyls and GPx activity were reduced in the CT1 group, while, in the CT2 group, the TBARS levels increased and GPx activity decreased (Table 3).

## 4. Discussion

Exercise is a strong ally in the treatment of obesity, and when performed with adequate intensity and frequency, it could provide protection against comorbidities of obesity. This includes improvement in the cardiovascular system (reduced blood pressure, increased peripheral blood flow, reduced atherosclerosis progression, and decreased oxygen demand of the myocardium), reduced risk of developing type 2 DM (by reducing IR), anxiety, and depression, and increased metabolic rate [18–20].

It is known that aerobic exercise is beneficial in weight loss, modulation of oxidative stress, and reduction of IR in obese individuals [21, 22]. But there are few studies applying the strength exercise associated with aerobic exercise (concurrent training) in this population. This training can help to reduce body weight and fat mass by increasing lean mass and basal metabolic rate [23–26].

These benefits were observed in this study, and both forms of concurrent trainings were able to reduce body weight and BMI. CT1 (5x/week) also reduced the body fat percentage and hip circumference and even increased basal metabolic rate. These findings were also shown in a study by Willis et al. [7] who demonstrated a reduction in weight, body fat percentage, and waist circumference in overweight adults who performed concurrent training 3x/week with an intensity of 65–80% VO_2peak_.

Another study also showed that overweight individuals who performed concurrent training for 12 weeks, 5 days/week, had a decrease in weight, BMI, body fat percentage, and waist circumference. However, in this same study, two other groups that performed strength training or aerobic training only had a decrease in waist circumference, showing the relevance of the type of training [6].

Our study also demonstrated a significant increase in free fat mass in CT1, which can result in neuromuscular adaptations such as increased muscle mass and maximal strength dynamic capacity and can improve basal metabolic rate in obese individuals. Also, other studies have shown that concurrent training has positive effects in the development of strength and muscle mass in both healthy young individuals [27] and overweight dyslipidemic individuals [28].

Furthermore, according to the IR data presented, the CT1 group showed an increase in fasting insulin level and HOMA-IR, although this increase was still within normal range (2–25 *μ*IU/mL). On the other hand, CT2 reduced glucose levels and HOMA-IR but did not alter fasting insulin. In the literature, there are few studies demonstrating the role of concurrent training in obese nondiabetics. But a study of subjects with metabolic syndrome and type 2 DM showed that concurrent training for 20 weeks at 70–80% VO_2peak_ reduced HOMA-IR as well as weight and waist circumference [29] starting on the third week, thereby corroborating our findings.

Studies have shown a direct relationship between exercise and insulin sensitivity [29, 30]. However, a short time of physical activity is associated with low insulin sensitivity, and a few days of rest are associated with increased IR [29–31]. Thus, regular exercise can improve insulin sensitivity in healthy subjects and obese nondiabetics [19, 32–34]. Notably, the positive effect of exercise on insulin sensitivity has been demonstrated 12–48 hours after the last session of exercise, but preexercise levels return in three or five days [35], highlighting the importance of regular exercise.

IR is associated with obesity, and both are related to oxidative stress. Clinical and animal models studies have shown that oxidative stress is a potential mediator of IR, particularly in skeletal muscle, because there is an inverse relationship between oxidative stress and insulin action [36–38]. Prolonged exposure to low levels of oxidative stress reduces insulin sensitivity (reduction in GLUT-4) [39] causing defects in its signaling [40, 41].

According to a study in a rat model of insulin-resistant obesity (Zucker), endurance training reduced protein oxidation (carbonyl) levels after training [36]. This research group also reported that this exercise improved insulin action and glucose transport in the soleus, plantar, and cardiac muscles [37]. These results are in line with our findings in concurrent training, that is, reduced damage to proteins (carbonyls). Furthermore, another study with Wistar rats showed a decrease in lipid peroxidation (TBARS), increase in antioxidant enzyme SOD activity, and reduction in triglyceride levels when concurrent training was performed 5x/week. It was also observed that this improvement in the antioxidant system was similar in the groups that performed exclusively aerobic or strength training [42].

Furthermore, the CT2 group showed lower GPx activity and a trend (*P* = 0.07) towards a reduction in CAT activity but no change in SOD activity and sulfhydryl level, which may have contributed to the increase in TBARS in this group. It is well known that exercise promotes increased oxidative stress, because during exercise the rate of oxygen consumption increases about 20 times, and in exhaustive exercise glutathione activity can be reduced [43]. Perhaps, the intensity and frequency of CT2 were not sufficient to modulate antioxidant defenses and thereby induce the necessary adjustments to reduce damage to lipids.

Many tissues can produce reactive oxygen species during physical exercise. Studies have investigated which organs are primarily responsible for reactive oxygen species production in exercising humans. The lack of in vivo studies on this topic is due to the difficulty of investigating the multifaceted nature of exercise, which involves several organ systems, and these organs are connecting through the increased energy requirement of contracting skeletal muscles [44]. Investigators have assumed that skeletal muscle provides the major source of free radical and reactive oxygen species generation during exercise [44]. Nonetheless, other tissues such as the heart, lungs, or blood may also contribute to the total body generation of reactive oxygen species during exercise [44, 45].

Studies have shown that prolonged or intense contractile activity alters the physiological environment in muscle fibers resulting in conditions that predispose these fibers to higher rates of reactive oxygen species production. Increased oxygen consumption in muscle fibers lowers intracellular oxygen tension during exercise, which can promote increased reactive oxygen species production [46]. Further, a rise in muscle temperature, increased CO_2_ tension, and decreased cellular pH are other exercise-related changes that can stimulate reactive oxygen species production in muscle. These contraction-induced changes can stimulate superoxide production at multiple subcellular sites [46, 47].

On the basis of this study, concurrent training may be an alternative to lowering weight and BMI, but when training is performed more frequently (5 times a week), these benefits can be enhanced by reducing body fat and protein damage as well. On the other hand, the effects of such training on oxidative stress parameters and IR were contradictory, and thus more studies are needed to elucidate the mechanisms of concurrent training in this population.

## Figures and Tables

**Table 1:** Schedule of concurrent training.

	Sessions					
	1–3^*^	4–9	10–15	16–21	22–24	25-26
Strength exercises						
Number of series		3	3	3	3	3
Number of reps		15	12	10	08	15
Intensity (% 1RM)		50%	60%	70%	75%	50%
Volume per session		45	36	30	24	45
Recovery time		60′′	90′′	90′′	90′′	60′′
Aerobic exercises						
Volume/time		30′	30′	30′	30′	30′
Intensity % VO_2peak_		50%	60%	70%	75%	50%
Stage Borg scale		12	13	14	15	12

^*^First 3 sessions to adapt to the exercises.

**Table 2:** Anthropometric parameters of obese individuals before and after concurrent training with different frequencies.

Anthropometric	CT1			CT2		
parameters	Before	After	*P*	Before	After	*P*
Body weight (kg)	96.06 ± 4.32	93.34 ± 4.26	<0.001	89.98 ± 4.64	87.20 ± 4.30	0.013
BMI (kg/m²)	36.73 ± 1.60	35.68 ± 1.55	<0.001	33.53 ± 1.03	32.55 ± 1.00	0.011
FFM (kg)	59.59 ± 2.90	61.74 ± 3.25	0.021	56.41 ± 3.68	57.27 ± 4.27	0.541
BF (%)	37.35 ± 1.57	33.78 ± 1.91	<0.001	38.11 ± 0.98	36.68 ± 1.39	0.062
WC (cm)	102.94 ± 2.94	101.91 ± 2.87	0.177	97.77 ± 2.55	95.11 ± 2.68	0.077
AC (cm)	113.27 ± 3.15	111.14 ± 2.79	0.074	105.33 ± 1.69	101.26 ± 1.25	0.026
HC (cm)	117.55 ± 2.27	113.23 ± 1.99	<0.001	113.79 ± 2.01	111.16 ± 1.80	0.007
BMR (kcal)	1825.82 ± 87.00	1872.36 ± 99.31	0.033	1715.77 ± 111.94	1735.54 ± 129.26	0.651

Variables presented as mean ± standard error. CT1: concurrent training 5 times per week; CT2: concurrent training 3 times per week. BMI: body mass index; FFM: free fat mass; BF%: body fat percentage, WC: waist circumference; AC: abdominal circumference; HC: hip circumference; BMR: basal metabolic rate.

*P* < 0.05 (significantly different). Paired *t*-test was utilized to compare before and after training in the same group.

**Table 3:** Comparison of markers of insulin resistance and oxidative stress in obese individuals before and after concurrent training.

	CT1			CT2		
	Before	After	*P*	Before	After	*P*
Insulin resistance markers						
Fasting glucose (mg/dL)	96.50 ± 3.85	85.66 ± 4.13	0.096	100.96 ± 3.76	85.69 ± 4.13	0.001
Fasting insulin (uUI/mL)	10.54 ± 0.84	14.29 ± 0.77	0.002	9.55 ± 0.77	8.54 ± 0.64	0.293
HOMA-IR	2.56 ± 0.26	3.77 ± 0.35	0.005	2.34 ± 0.56	1.79 ± 0.52	0.009
Oxidative stress markers						
TBARS (nmol/mL)	3.69 ± 0.31	3.69 ± 0.11	0.987	4.70 ± 0.17	6.35 ± 0.049	0.015
Carbonyl (nmol DNPH/mg)	116.36 ± 18.36	34.45 ± 7.46	0.012	24.96 ± 9.67	24.39 ± 8.57	0.965
Sulfhydryl (*µ*M/mg)	11.83 ± 11.03	9.03 ± 1.23	0.145	5.20 ± 1.04	4.09 ± 0.41	0.169
CAT (UCAT/mg)	2.79 ± 0.66	4.73 ± 1.24	0.281	3.26 ± 0.63	1.83 ± 0.28	0.074
SOD (USOD/mg	1.85 ± 0.50	2.40 ± 0.86	0.667	1.29 ± 0.46	3.07 ± 1.38	0.235
GPx (UGPx/mg)	3.86 ± 0.40	3.37 ± 0.37	<0.001	4.57 ± 0.45	4.22 ± 0.42	0.002

Variables presented as mean ± standard error. CT1: concurrent training 5 times per week; CT2: concurrent training 3 times per week. HOMA-IR: homeostasis model of insulin resistance; TBARS: thiobarbituric acid reactive substances; CAT: catalase activity; SOD: superoxide dismutase activity; GPx: glutathione peroxidase activity.

*P* < 0.05 (significantly different). Paired *t*-test was utilized to compare before and after training in the same group.
